# Renoprotective effects of metformin

**DOI:** 10.1186/2008-2231-21-36

**Published:** 2013-05-16

**Authors:** Running Hamid Nasri

**Affiliations:** 1 Department of Nephrology, Division of Nephropathology, Isfahan University of Medical Sciences, Isfahan, Iran

## Abstract

Metformin as a biguanid drug entered to the market 50 years ago and now is generally recommended as the first-line treatment in type 2 diabetes, especially in overweight patients, however in recent years new indications for its use have emerged . It improves peripheral and liver sensitivity to insulin, reduces basal hepatic glucose production, increases insulin-stimulated uptake and utilization of glucose by peripheral tissues, decreases hunger and causes weight reduction.Recently, much attention has been made toward the possible kidney protective efficacy of metformin. Recent studies have proven that metformin, possesses antioxidant properties, too.

## 

Dear Editor-in-Chief,

Metformin as a biguanid drug entered to the market 50 years ago and now is generally recommended as the first-line treatment in type 2 diabetes, especially in overweight patients, however in recent years new indications for its use have emerged [1-3]. It improves peripheral and liver sensitivity to insulin, reduces basal hepatic glucose production, increases insulin-stimulated uptake and utilization of glucose by peripheral tissues, decreases hunger and causes weight reduction [1,3,4]. Recently, much attention has been made toward the possible kidney protective efficacy of metformin. Recent studies have proven that metformin, possesses antioxidant properties, too [1,5]. Reduction of apoptosis, induced by oxidative stress, in endothelial cells and prevention of vascular dysfunction was found with metformin treatment [1,5,6]. Previously Morales et al. showed that gentamicin-induced renal tubular damage is attenuated by metformin [7]. To better evaluate the ameliorative effect of metformin against gentamicin tubular toxicity, we conducted a study on male Wistar rats [8]. In this study, we found, the preventive property of metformin on gentamicin-induced acute kidney injury. Hence, it might be beneficial in patients under treatment with this drug [8]. Recently, Taheri et al., found, the ameliorative property of metformin against unilateral ischemia–reperfusion induced injury in rats [9], which is in accord with our findings. More recently, to test the efficacy of co-administration of garlic extract and metformin for prevention of gentamicin–renal toxicity in Wistar rats, we conducted another study on 70 male rats [10]. The result of this study indicates that metformin and garlic or their combination has both curative and protective effects against gentamicin nephrotoxicity. Hence, garlic extract could safely be used together with metformin to increase the antioxidant potency to ameliorate gentamicin-tubular toxicity [10]. The well-known enzyme, AMP-activated kinase (AMPK), is associated with the pleiotropic actions of metformin [11]. This enzyme regulates cellular and organ metabolism [5,6,11]. AMPK is a phylogenetically conserved serine/threonine protein kinase imagined as a fuel gauge monitoring systemic and cellular energy condition [5,6,11] and plays an important role in protecting cellular functions under energy-restricted circumstances [5,6,11]. Various data indicates that AMPK activation by metformin is secondary to its effect on the mitochondria as the primary target of this agent [5,6,11]. Recent findings have revealed the mitochondrial effects of metformin [5,6,11,12]. Indeed, there is evidence that, when it is used alone, the advantageous effect of metformin may be due to its mild inhibition of the mitochondrial respiratory chain [5,6,11,12]. It is also evident that metformin treatment, significantly attenuates the increase in malondialdehyde and total reactive oxygen species generation and restores the decrease in both enzymatic and non-enzymatic antioxidants [5], thus, poses the ameliorative effects against toxic effects to the renal tubules [6,11-14], as we observed in the mentioned studies. However, the main question is, whether these experimental findings are applicable in clinical studies. We are mostly unanimous to use metformin as a first-line glucose-lowering agent [14-19]. However, it cannot be given to a proportion of patients with type 2 diabetes due to various contraindications that could lead to an increased risk of lactic acidosis [1,2,16,17]. Scientists emphasize that it must be used with caution in estimated glomerular filtration rates of below 60 mL/minute and discontinued when estimated glomerular filtration rate is less than 30 mL/minute [1,2,16,17]. Metformin-associated lactic acidosis is a severe metabolic disorder with high mortality and in severe cases patients may need renal replacement therapy [19]. However, risk of metformin-associated lactic acidosis could be decreased by avoiding metformin use in patients with high risk of sepsis, renal impairment, hypovolemia, reduced kidney capacity such as old age patients [19]. Nevertheless, in these conditions, metformin may indeed act as an 'innocent bystander' [1,2,16,17,19]. A recent review by Papanas et al. remarking on the relationship between metformin and cardiac insufficiency revealed that metformin might even reduce the risk of cardiac failure morbidity and mortality in diabetics [20]. To find the advantage of adjunct metformin and insulin therapy in the management of glycemia in critically ill patients, Mojtahedzadeh et al. studied thirty three traumatized adult patients who were admitted to the hospital. Patients were randomly assigned to receive one of three protocols including intensive insulin monotherapy (A), metformin monotherapy (B), and intensive insulin therapy in combination with metformin (C) to maintain blood glucose level between 80–120 mg/dl. They found that metformin was able to reduce insulin requirements in glycemic management of critically ill patients independent of its plasma concentration. They concluded that metformin was effective to reverse insulin resistance without induction of lactic acidosis [21]. On the other hand, it is possible that the use of metformin would be favorable in many with chronic renal failure according to the advantages linked with lessening of metabolic syndrome and cardiovascular protection. The actuality of severe metformin-induced lactic acidosis in the absence of chronic kidney failure raises the question of limitation of its use in these patients [16,20]. Diabetic nephropathy is one of the most important complications of diabetes mellitus [22-26] and metformin has been widely used for the treatment of type 2 diabetes [17-19]. Kim et al. conducted a study using metformin for spontaneously diabetic rats for 17 weeks. They found that treatment of diabetic rats with metformin restored podocyte loss. They suggested that diabetes-induced podocyte loss in diabetic nephropathy could be suppressed by metformin, through the repression of oxidative injury [27]. Thus according to our results and those published by previous investigators, metformin protects against tubular injury by restoring the biochemical alterations and modulation of oxidative stress on the tubules. Furthermore, according to the study of Kim et al., metformin protects podocytes in diabetic nephropathy. While in diabetic nephropathy, there is also tubular cell injury [28-31] due to glycosuria. These findings can more potentiate the clinical use of metformin in the prevention of diabetic nephropathy [32-36]. In this regard, to understand the metformin kidney protective properties better, more experimental rat model or clinical studies are suggested.

## Competing interest

The author declared no competing interests.
